# Distinct bacterial communities affiliated with two types of shredder-produced particles in streams

**DOI:** 10.1093/femsec/fiaf091

**Published:** 2025-09-16

**Authors:** Pratiksha Acharya, Mourine J Yegon, Christian Griebler, Simon Vitecek, Katrin Attermeyer

**Affiliations:** WasserCluster Lunz – Biological Station, Dr. Carl Kupelwieser-Prom. 5, 3293 Lunz am See, Austria; Department of Functional and Evolutionary Ecology, Unit Limnology, University of Vienna, Djerassiplatz 1, 1030 Vienna, Austria; WasserCluster Lunz – Biological Station, Dr. Carl Kupelwieser-Prom. 5, 3293 Lunz am See, Austria; Institute for Hydrobiology and Water Management (IHG), University of Natural Resources and Life Sciences, Gregor-Mendel-Straße 33/DG, 1180 Vienna, Austria; Department of Functional and Evolutionary Ecology, Unit Limnology, University of Vienna, Djerassiplatz 1, 1030 Vienna, Austria; Institute for Hydrobiology and Water Management (IHG), University of Natural Resources and Life Sciences, Gregor-Mendel-Straße 33/DG, 1180 Vienna, Austria; Department of Ecology, University of Innsbruck, Technikerstraße 25, 6020 Innsbruck, Austria; WasserCluster Lunz – Biological Station, Dr. Carl Kupelwieser-Prom. 5, 3293 Lunz am See, Austria; Department of Functional and Evolutionary Ecology, Unit Limnology, University of Vienna, Djerassiplatz 1, 1030 Vienna, Austria

**Keywords:** leaf conditioning, leaf litter decomposition, microbial assemblage, microbial community composition, stream

## Abstract

Leaf litter decomposition is a vital ecosystem process in which macroinvertebrate-shredders produce substantial amounts of fine particulate organic matter (FPOM) via sloppy feeding and defecation, creating a substratum and substrate for microbial assemblages. However, microbial communities colonizing the shredder-produced FPOM are understudied compared to those in streams and on original leaves. Here, we investigated the bacterial community composition on shredder-produced FPOM in a laboratory experiment. We fed alder, beech, and maple leaves conditioned under oxic or anoxic conditions to *Sericostoma* (Insecta: Trichoptera) larvae. We collected shredded leaf particles and faecal pellets as shredder-produced FPOM at different times and examined their microbial communities using 16S rRNA amplicon sequencing. We hypothesized that shredder-produced FPOM types harbor diverse, distinct, and specialized microbial taxa in response to leaf species and conditioning. We found significantly higher alpha diversity on shredded leaves compared to faecal pellets. Microbial communities on faecal pellets differed from initial leaf communities and with anoxic and oxic conditioning. Bacterial communities developing on leaves were dominated by common leaf decomposers including *Flavobacterium* and *Pseudomonas* whereas faecal pellets harbored gut bacterial taxa including *Acinetobacter* and *Carnobacterium*. These results underline the importance of conditioning and shredder activity in shaping FPOM-attached bacterial communities, increasing bacterial diversity in stream ecosystems.

## Teaser

Shredder-derived fine particulate organic matter hosts distinct bacterial communities influenced by conditioning and gut passage, highlighting its role in enhancing microbial diversity in stream ecosystems.

## Introduction

Microbial communities are key to the decomposition of organic matter in freshwater ecosystems (Webster and Benfield 1986, Allan et al. 2021). In headwater streams, the input of leaves and their decomposition are significant processes in carbon cycling (Vannote et al. 1980, Webster and Benfield 1986). Microorganisms including fungi and bacteria colonize and decompose leaf litter and their growth results in the accumulation of nutrients in the litter (Findlay et al. 2003, Mehring et al. 2015), a process known as “conditioning” (Bärlocher and Kendrick 1975, Gessner et al. 1999, Gonçalves et al. 2024). Over time, physical abrasion and macroinvertebrate consumers (i.e. shredders) further fragment the large leaf litter or coarse particulate organic matter (CPOM) via sloppy feeding and defecation (Wallace and Webster 1996, Graça 2001), ultimately producing different types of fine particulate organic matter (FPOM), such as shredded leaf particles and faecal pellets. FPOM is a crucial resource for collectors and deposit-feeding organisms (Bundschuh and McKie 2016) and also harbors a diverse and active microbial community (Acharya et al. 2025) that can influence in-stream leaf litter breakdown and energy cycling. Understanding the link between community composition and functions is essential for elucidating these processes and the presence of specific taxa provides insight into the biochemical and physiological activities occurring during decomposition (Gessner et al. 2010).

Leaf-associated microbial communities are shaped by their surrounding environment (Gonçalves et al. 2024). Generally, microbial communities in streams respond to environmental conditions, such as hydrology (i.e. flow conditions and water retention), biotic interactions (i.e. microbial competition and predator–prey interactions), nutrient gradients, and physical and chemical parameters, such as temperature, pH, or dissolved oxygen (Cummins 1974, Petersen and Cummins 1974, Savio et al. 2015, Luo et al. 2020, Ma et al. 2020, Huang et al. 2022, Stadler and Del Giorgio 2022, Guo et al. 2023, Karačić et al. 2024, Sadeghi et al. 2024). For instance, oxygen concentrations can vary widely in stream microhabitats: Leaves in fast-flowing or turbulent oxygen-rich microhabitats are potentially decomposed quickly by aerobic microbial communities that include prokaryotes but also eukaryotes and follow a range of oxidative metabolic pathways (Brune et al. 2000, Karačić et al. 2024, Khomutovska et al. 2024). In contrast, large amounts of leaves can also accumulate in thick packs in slow-flowing sections or stagnant pools (Wantzen and Wagner 2006, Foucreau et al. 2013). These are frequently hypoxic or anoxic microhabitats dominated by specialized bacterial communities, such as anaerobic or facultative anaerobic taxa that mostly follow fermentation pathways (Degelmann et al. 2009). Differences in oxygen availability affect microbial community assembly and composition. In combination with the properties of the original leaf litter, this has potential consequences for leaf litter decomposition (LLD) rates and efficiency (Gessner 2010, Dieter et al. 2011). While the effects of environmental conditions, such as oxygen availability on benthic and pelagic microbial communities are relatively well-known, their influence on leaf-associated microbial communities remains to be investigated.

Fungi are amongst the first colonizers of abscised leaves in the stream ecosystem with a predominant role at the early stages of LLD whereas bacteria usually dominate the decomposition process at a later stage (Gessner and Chauvet 1994, Gessner and Schmitt 1996, Foucreau et al. 2013, Pascoal et al. 2021). However, specific types of bacteria, including members of the genera *Pseudomonas, Sphingomonas, Pedobacter*, and *Massilia* are also found on newly submerged leaves where they support LLD by degrading recalcitrant polymers, such as lignin, cellulose, and hemicellulose (Tláskal et al. 2016, Silverman et al. 2017, Khomutovska et al. 2024). Indeed, some bacterial taxa must be considered ecological specialists, such as *Massilia* (nitrogen reducer; Hou et al. 2017), *Sphingomonas* (chitin degrader; Tláskal et al. 2016), and Alphaproteobacteria (phenol and tannin degrader; Wymore et al. 2016). On decomposing leaves, Wymore et al. (2016) found that *Bacteroidetes* and Betaproteobacteria dominated the bacterial assemblage, while Verrucomicrobia, along with Alphaproteobacteria and Gammaproteobacteria, became more abundant over time. The primary factor driving variation in bacterial composition was time whereas leaf species physiochemistry also significantly contributed to explaining the observed variations. These results show that the bacterial community on decomposing leaves in streams can be highly variable over time but also influenced by leaf species via differences in their chemical composition. Furthermore, shredder-produced FPOM can provide distinct ecological niches for bacterial taxa compared to the original CPOM due to changes in substrate quality or surface area via shredders gut passage or shredding. Therefore, the bacterial communities in shredder-produced FPOM may differ from those on the original leaf litter, potentially altering the nutritional value of the organic matter (Gessner et al. 1999), and playing distinct roles in organic matter turnover (Frossard et al. 2013) and nutrient cycling (Kominoski et al. 2009) within the stream’s food web. Given the critical role of bacteria during LLD and associated energy cycling, understanding the presence and functional contributions of specific bacterial taxa during decomposition is essential. In this context, data on individual taxa can provide valuable insights into the biochemical and physiological processes driving litter breakdown. While substantial research targeted the initial microbial colonization and its role in LLD, the bacterial diversity and community composition of shredder-produced FPOM have received less attention.

Shredded leaves, one type of shredder-produced FPOM, may exhibit bacterial community patterns similar to those of intact leaves. However, the effects of shredder gut passage on the bacterial composition in faecal pellets, the other form of shredder-produced FPOM, remain largely unexplored. Leaf litter undergoes significant microbial transformations during gut passage and only specialist or resilient taxa typically survive the passage (Plante et al. 2023). As shredders consume and fragment leaf litter, the microbial assemblages on the ingested material are exposed to gut conditions, such as digestive enzymes, changes in pH, and potentially anoxic conditions, which can selectively promote or inhibit certain bacterial taxa. Research on various deposit-feeding taxa, including Polychaeta (Plante and Wilde 2004), echinoids (Gao et al. 2014), hemichordates (King 2018), and crustaceans (Mongkol et al. 2018), has demonstrated that gut passage alters bacterial community composition on defecation products, likely through selective digestion (Plante et al. 1996, King 2018) or seeding from resident gut microbiomes (Lau et al. 2002, King 2018, Dale et al. 2019). This process could play a crucial role in shaping the diversity and composition of bacterial communities associated with FPOM in streams, including changes in nutritional quality and bioavailability of FPOM to other stream consumers (Bundschuh and McKie 2016) and thus influence downstream nutrient cycling (Cheever et al. 2013, Frossard et al. 2013).While numerous studies have explored the gut microbiomes of aquatic insects, including stream-dwelling species, research specifically addressing the influence of the gut passage in freshwater shredders involved in LLD is limited. Notably, there is a lack of studies examining the bacterial communities attached to the faecal pellets of shredders, highlighting a key gap in understanding their role in bacterial succession and nutrient cycling within freshwater ecosystems.

In this study, we aim to assess the variation in bacterial assemblages on leaf litter under different environmental conditions (i.e. oxygen level) and on leaf litter before ingestion and on faecal pellets after passage through the digestive tract (gut passage) of *Sericostoma* sp., using DNA metabarcoding. Specifically, our aim was to evaluate how leaf species, leaf conditioning under different oxygen levels, and gut passage affect the diversity and composition of bacterial communities associated with shredder-produced FPOM in a microcosm feeding experiment and identify indicative bacterial taxa dominating shredder-produced FPOM during litter breakdown. We hypothesized that (i) the oxic conditioned leaves have different and more diverse bacterial community than anoxic conditioned leaves and (ii) two types of shredder-produced FPOM—mechanically shredded leaves from sloppy feeding and faecal pellet FPOM—would be colonized by distinct bacterial communities due to selective digestion and microbial filtering in the gut (Plante et al. 1996, Aira et al. 2022), which we moreover expected to vary with leaf species and prior leaf conditioning.

## Materials and methods

### Preparation for laboratory feeding experiment

We collected naturally abscised alder, beech, and maple leaves around Lunz am See, Austria, dried and stored them in the dark at room temperature. For the experiment, we cut the leaves into 18 mm diameter circular discs prior to microbial colonization. For microbial colonization under anoxic conditions, we buried the leaf discs of each leaf species packed in mesh bags (1 mm mesh size) in a 1:1 clay (Glorex AG, Füllinsdorf, Switzerland) slurry. We mixed the clay slurry with stream water from Oberer Seebach (OSB; 47° 51′ N, 15° 04′ E), allowing the clay to settle and cover the discs within a short period, and leaving them undisturbed for 4 weeks. We confirmed anoxic conditions by measuring dissolved oxygen concentrations directly above the clay layer four times during the conditioning period. Leaves were washed immediately before use in experiment with water from the same stream to remove excess sediments. For microbial colonization under oxic conditions, we submerged the leaf discs in freshly collected, aerated stream water for 1 week. We confirmed the oxic conditions by regularly monitoring the dissolved oxygen concentration in the water column (WTW Oxi 315i; Xylem Analytics, Weilheim, Germany). Samples of oxic and anoxic conditioned leaves were taken right after conditioning but before the start of the feeding experiment to assess the initial microbial communities. We refer to these leaves as mechanically shredded leaves at the initial stage of the feeding experiment (“Initial shredded leaves”). More details of the experiment preparation can be found in Acharya et al. (2025).

A day before the start of the feeding experiment, we collected the aquatic shredder species, *Sericostoma* sp. (Trichoptera; larval stage; hereafter referred to as “larvae”) of similar size (head capsule width 2.0 ± 0.1 mm) from Unterer Seebach (USB; 47° 51′ 29″ N 15° 2′ 4.74″ E) and transported them to the laboratory. We acclimated them in separate food-grade plastic cups [polylactic acid (PLA), volume: 4 cl] at 12°C in filtered stream water (0.7 µm, 450°C, 4 h precombusted Whatmann GF/F filters), and they were starved for 24 h before the start of the feeding experiment.

### Microcosm setup

We conducted a 25-day feeding experiment at 12°C and a light: dark cycle of 11:13 h in a full-factorial design. We set up five replicates of each of the six treatments that consisted of the three leaf species and the two oxic–anoxic conditioning treatments, resulting in a total of 30 microcosms. Each microcosm consisted of a white food grade plastic bucket (1100 ml volume and 122 mm bottom diameter) with a steel mesh (pore size 1 mm) at a height of 4.5–5 cm from the bottom to allow shredder-produced FPOM to pass through. We adapted the microcosm setup from a previous study (Rubio-Ríos et al. 2022). We supplied organic matter free sand particles (muffle furnace, 450°C, 4 h precombusted) in aluminum Petri dishes for shredders to make their cases. We added 10 shredders to each microcosm and fed them with the conditioned leaves of one of the three species. During the 25-day feeding experiment, we collected and analyzed bacterial community composition on the larval faecal pellets at three time points: on 5th, 15th, and 25th day (5 d, 15 d, and 25 d). From each treatment, we transferred each larva to a single food-grade plastic cup (polylactic acid or polylactide—PLA) filled with 20 ml of 0.7 µm-filtered stream water and left them to evacuate their gut contents for 24 h; thus, we only collected faecal pellets and no shredded leaf particles. We collected the faecal pellets from each plastic cup using a Pasteur pipette after returning the larvae to the same microcosm and after replacing the water in the microcosms with freshly collected stream water (see details of microcosms setup in Acharya et al. 2025).

At the end of the experiment, we collected the remaining leaf discs that were partly eaten by the shredders in order to assess the final microbial communities established on leaves and stored them at −80°C until further processing. We refer to these leaves as “Final shredded leaves.”

### DNA extraction

For DNA extraction, we initially aimed to pool faecal pellets from two larvae per microcosm. However, our tests revealed that this amount was insufficient for DNA extraction, and we thus pooled the faecal pellets from two larvae per microcosm from all five replicates per treatment (faecal pellets per 2 larvae × 5 microcosm per treatment = faecal pellets from 10 larvae). This resulted in just one sample per treatment per timepoint for the analysis (*n* = each treatment × 3 sampling timepoints). We stored the samples for the DNA extraction at −80°C until further processing.

We extracted total genomic DNA from freeze-dried (Virtis™ Genesis Freeze Dryer, for minimum 24 h) leaves (*n* = 36, 3 replicates × 6 treatment × 2 timepoints) and faecal pellets ( = 18, 6 treatment × 3 sampling timepoints) using the DNeasy PowerSoil Pro kit (Qiagen, Hilden, Germany) following the manufacturers’ protocol (leaves were first cut into smaller pieces with a sterile scalpel). DNA concentrations were quantified fluorometrically with the Qubit™ 1X dsDNA HS Assay Kits using an Invitrogen Qubit 4.0 Fluorometer (Thermo Fisher Scientific).

### Amplicon sequencing

We used the extracted total DNA to amplify and sequence the V4 region of the 16S rRNA gene. To this end, the primer pair 515F (GTG YCA GCM GCC GCG GTA A) and 806R (GGA CTA CNV GGG TWT CTA AT) (Apprill et al. 2015, Parada et al. 2016) was used for amplification; barcoding and adapter ligation were achieved in a second step as described in Pjevac et al. (2021)for sequencing on an Illumina MiSeq platform with 600-cycle v3 chemistry (2 × 300 bp paired-end reads). Raw sequencing data were processed using a FASTQ workflow (Basespace, Illumina) with default settings, and sorted into unique amplicon sequence variants (ASVs) using the DADA2 pipeline (Callahan et al. 2016) in R (R core team 2023) using the recommended workflow. FASTQ reads were trimmed at 220/230 nt with allowed expected errors of two-fourths. The representative ASVs generated were subsequently merged and classified using the SILVA r138.1 database using default parameters (Quast et al. 2012), and taxonomy assigned using the assignTaxonomy() function in DADA2 with a confidence threshold of 0.05 for prokaryotic 16S rRNA gene amplicons.

### Data filtering and statistical analysis

Before statistical analysis, we removed ASVs representing Eukaryota and Archaea, as well as ASVs classified as mitochondria or chloroplasts, as well as singleton and doubleton ASVs. Further, ASVs that did not have phylum-level information were also removed. Finally, we combined ASV read abundances, taxonomy, and sample metadata into a single dataset (function *phyloseq; phyloseq* package) (McMurdie and Holmes 2013) in R for subsequent analyses. All data analyses and visualizations were done in R 4.2.3 version (R core team 2023).

To test diversity and compositional differences of bacterial communities between shredded leaves and faecal pellets, we calculated alpha diversity and beta diversity. We used species accumulation curves to compare estimates of alpha diversity from each FPOM type (function *specaccum; vegan* package; Oksanen et al. 2022). We compared species richness (Observed and Shannon–Wiener Index) metrics on the rarefied data to the lowest sequencing effort (1000 sequences) (function *estimate_richness; phyloseq* package). After calculating Shannon Diversity Index, we ran a linear model where leaf species, conditioning, FPOM types, and their interactions were used as fixed factors. We then evaluated the model fit by running Analysis of Variance (Type III ANOVA) and verified the assumptions of normality and homoscedasticity by plotting residuals. Afterwards, we calculated the effect size (function *effectsize; effectsize* package) (Ben-Shachar et al. 2020) to identify the strongest influencing fixed factor. We did not conduct rarefaction for any other analysis, and other transformations were applied to standardize data without losing information.

We calculated beta diversity on filtered and standardized data tables using Hellinger’s transformation on ASV read abundances (nonrarefied matrices) (function *decostand; vegan* package). We assessed the bacterial community structures in the samples using nonmetric multidimensional scaling system (NMDS; function *ordinate; phyloseq* package) based on Bray–Curtis distance matrix of transformed ASVs abundances (function *vegdist; vegan* package). The goodness of fit of the NMDS is given by stress values. Stress values above 0.2 might indicate unreliable ordinations. We compared bacterial community composition among FPOM types, leaf species and between conditioning by permutational multivariate analysis of variance (PERMANOVA; function *adonis2; vegan* package) on the transformed ASVs, using “Bray–Curtis” dissimilarities as a distance measure. The assumption of homogeneous within-group dispersion was tested (function *betadisper; vegan* package) and was fulfilled for all groups.

For our second hypothesis regarding the differences in taxonomic composition, we did a Kruskal–Wallis test, followed by Wilcoxon rank-sum test for pairwise comparisons of mean of relative sequence abundance of specific taxonomic groups (e.g. phylum and class) among FPOM types (function *compare_means; ggpubr* package). *P*-values were reported after Benjamini–Hochberg (BH) correction (Benjamini and Hochberg 1995). In addition, to identify the group of samples with similar bacterial community composition, we performed a hierarchical clustering analysis on relative abundance of count taxon of ASVs using Euclidean distance and Ward’s linkage (function *dist; stats* package), that minimizes the sum of squares of any two clusters (function *as.dendrogram; stats* package). Further, to assess the differences in the specialized taxa among the distinct sample groups, ISA (function *multipatt; Indicspecies* package) was applied on relative sequence abundance of bacterial community composition. To retain meaningful taxa from each group, abundant taxa with a *P*-value above 0.05 were excluded after *P*-value adjustment with BH false discovery rate method. Since the number of significant species was still too big, we selected the 15 taxa with the highest association of each group.

## Results

### Taxonomic composition of bacterial communities in shredder-produced FPOM

**Figure 1.**
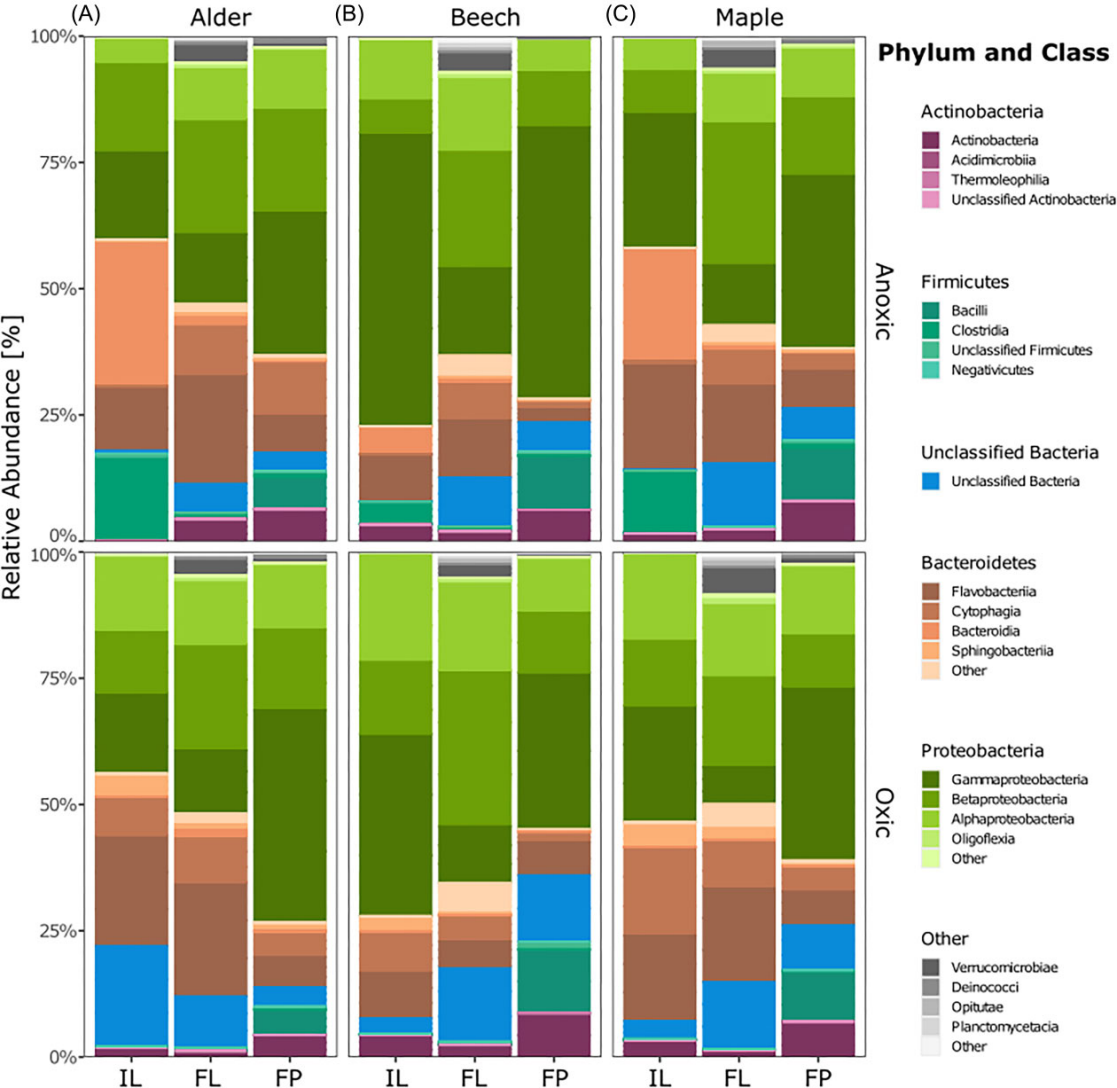
Taxonomic composition and relative abundance of individual taxonomic groups for each FPOM type and associated with leaf treatments: alder (A), beech (B), and maple (C). Additionally, the graph shows the data for anoxic- (top) and oxic-conditioned leaves (bottom). Each phylum is indicated by a different color and each class is indicated by a different shade of the color. Only the five most abundant phyla and four most abundant classes across phyla level for each treatment are shown whereas the remaining phyla and classes are grouped as “Other.” Abbreviations: IL; initial leaves, FL; final leaves, and FP; faecal pellets.

The taxonomic composition of bacterial communities based on DNA extracted from initial and final shredded leaves, and faecal pellets is visualized in Fig. 1 and Fig. S1. The relative abundances of specific taxonomic groups (at phylum and class level) differed significantly among the two shredder-produced FPOM types (Kruskal–Wallis, *P* < 0.05; Tables S1 and S2). However, the majority of the abundant taxa at phylum and class level did not differ between the two conditioning and among the three leaf species (Kruskal–Wallis, *P* > 0.05; Fig. 1 and Tables S3–S6). In general, taxonomic classification of the obtained ASVs using the Silva database showed that all samples were dominated by ASV sequences assigned to the phyla Proteobacteria (on average 55% in initial shredded leaves, 51% in final shredded leaves, and 61% in faecal pellets), followed by Bacteroidetes (on average 32% in initial leaves, 29% in final leaves, and 12% in faecal pellets), Firmicutes (on average 5.9% in initial shredded leaves, 0.7% in final shredded leaves, and 10% in faecal pellets), Actinobacteria (2.5% in initial shredded leaves, 2.2% in shredded final leaves, and 7.3% in faecal pellets), and Verrucomicrobia (0.03% in initial shredded leaves, 5.1% in shredded final leaves, and 0.6% in faecal pellets) (Fig. 1 and Fig. S1; Table S7). At class level, we found that the initial shredded leaves were dominated by Gammaproteobacteria (30%), Flavobacteriia (15%), and Alphaproteobacteria (13%), whereas final shredded leaves were more dominated by Betaproteobacteria (24%), followed by Flavobacteriia (16%), and Alphaproteobacteria (14%). As with the initial communities on shredded leaves, bacterial communities on faecal pellets were dominated by Gammaproteobacteria (36%), but then followed by Betaproteobacteria (14%), Alphaproteobacteria (11%), and Bacilli (9.1%) (Fig. 1; Table S7).

### Bacterial taxa richness and Shannon diversity in shredder-produced FPOM

**Figure 2.**
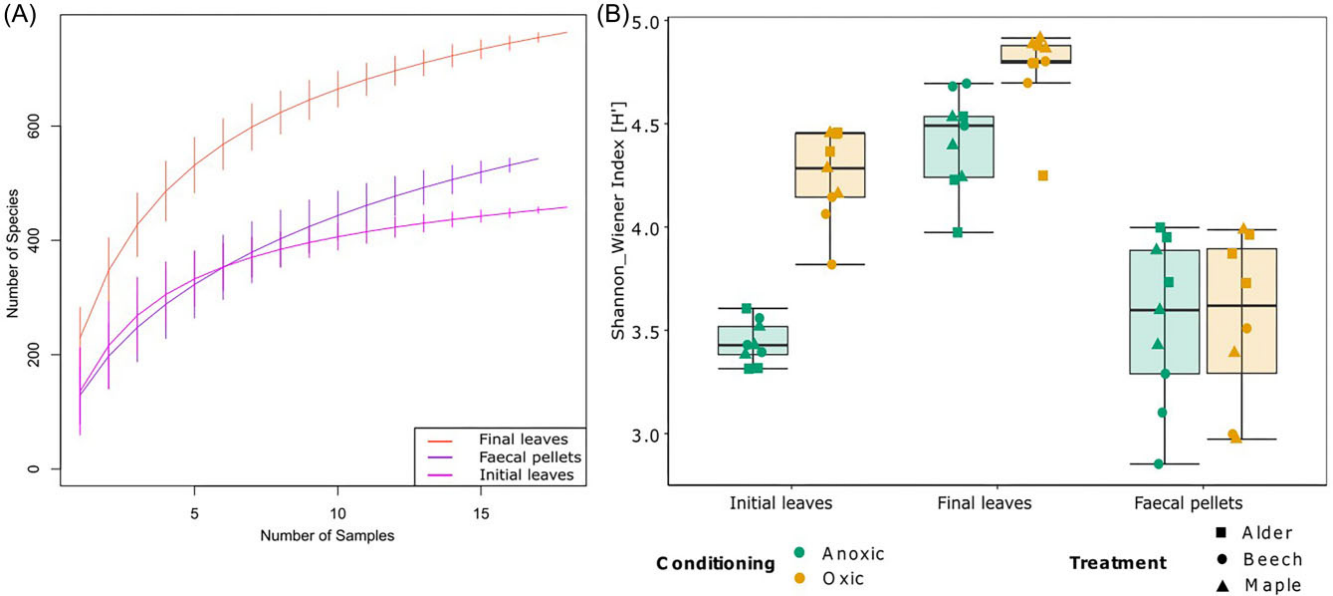
Species accumulation curves based on rarefaction method (A) and boxplot of Shannon bacterial diversity (B) across different FPOM types collected during 25 days of laboratory feeding experiment. In figure A, the vertical line on each curve indicates the error bar around the mean number of species observed per number of samples. The curves indicate final shredded leaves, faecal pellets, and initial shredded leaves. In figure B, two colors distinguish the samples from oxic or anoxic conditioning and three symbols indicate alder, beech, and maple treatments. The boxplots visualize the median of each FPOM type (line), the first and third quartiles (hinges), the 1.5 * interquartile ranges (whiskers).

For the quality assessment of our sequencing dataset, we rarefied the data to the lowest sequencing effort (1000 sequences). We plotted rarefied species accumulation curves for the three different types of FPOM collected during the experiment: initial shredded leaves, final shredded leaves, and faecal pellets, as a function of the sample number (Fig. 2A). Rarefied species accumulation curves indicated good coverage of richness in our samples but approached a quasi-horizontal asymptote only in the case of initial leaves (Fig. 2A). Richness estimates for the faecal pellets and especially final leaves were steeper, indicating a potentially greater richness than covered by our analyses. Overall, the final shredded leaves had the highest species richness (mean ± standard deviation; 229 ± 27 taxa) while that of faecal pellets and initial shredded leaves were considerably lower with 129 ± 26 and 136 ± 39 taxa, respectively.

Additionally, we calculated the Shannon Diversity Index on the rarefied dataset that was again highest in the final shredded leaves. The index ranged from 4.49 ± 0.24 (anoxic) and 4.81 ± 0.19 (oxic) in final shredded leaves to 3.43 ± 0.10 (anoxic) and 4.29 ± 0.20 (oxic) in initial shredded leaves and 3.60 ± 0.39 (anoxic) and 3.61 ± 0.40 (oxic) in faecal pellets (Fig. 2B). For this parameter, the FPOM type, leaf species, and the interactions between leaf species and FPOM types and between conditioning and FPOM types were significant (Table 1). Specifically, the FPOM type “Final leaves” showed a significant positive effect on Shannon diversity compared to the reference FPOM type “Faecal pellets” (linear model: Estimate = 0.36, *P* < 0.05; see Table S9 for all effect sizes), while “Initial leaves” had a significant negative effect (−0.45, *P* < 0.05). Among leaf species, beech had a significantly reduced Shannon diversity compared to the reference alder (−0.79, *P* < 0.05). Finally, the overall conditioning effect was not significant (Table 1). However, resulting from the significant interaction between conditioning and FPOM type, we found that for “Initial leaves” the oxic conditioning significantly increased Shannon diversity compared to anoxic conditioning (1.00, *P* < 0.05) with ~1.3 times higher Shannon diversity (Table S8).

**Table 1.** Effects of leaf species, conditioning, FPOM type, and their interactions on bacterial diversity of samples during feeding experiment based on linear model analysis. Abbreviations; St: Standardized, SS: sum of square, and df: degrees of freedom.

Factors	St. coefficient	SS	df	*F*-value	*P*-value
FPOM type	0.38	0.999	2	10.915	**<0.001**
Conditioning	0.001	0.001	1	0.031	0.860
Leaf species	0.38	0.968	2	10.583	**<0.001**
FPOM type * Conditioning	0.32	0.747	2	8.162	**0.001**
FPOM type * Leaf species	0.41	1.121	4	6.128	**<0.001**
Conditioning * Leaf species	0.05	0.081	2	0.888	0.421
FPOM type * Conditioning * Leaf species	0.14	0.252	4	1.380	0.261
Residuals		1.601	35		

Statistically significant *P*-values (< 0.05) are shown in bold.

### Structure of bacterial community composition in shredder-driven FPOM

**Figure 3.**
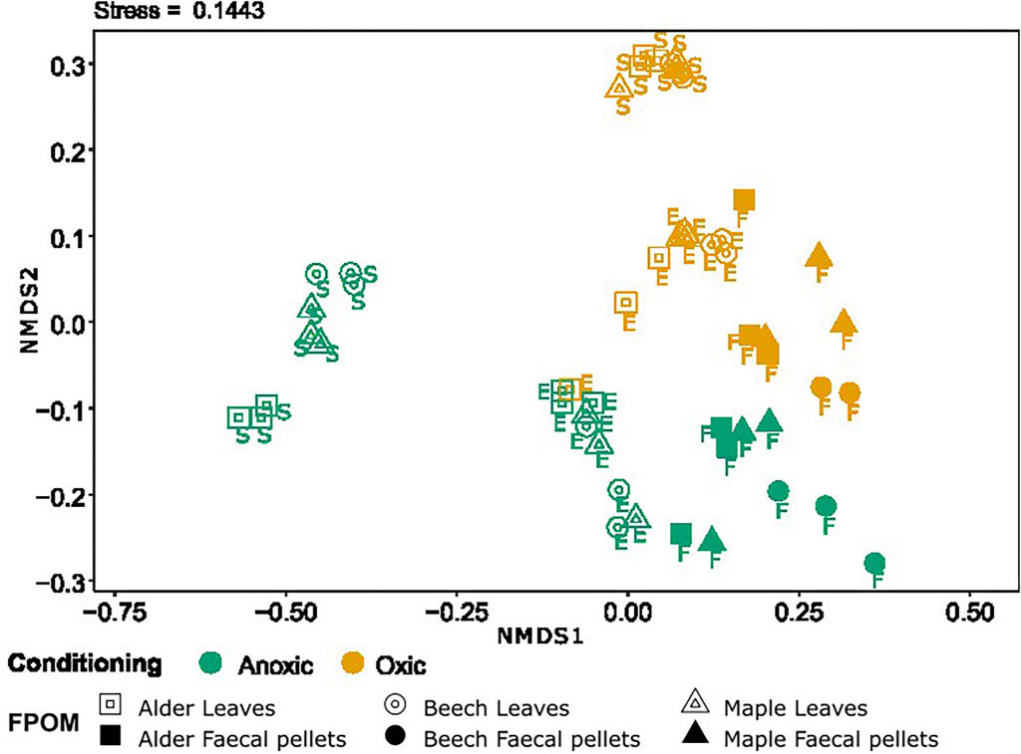
NMDS showing the clustering of normalized and Hellinger-transformed 16S sequencing data based on Bray–Curtis distances of bacterial communities associated with initial leaves, final leaves, and faecal pellets. Abbreviation: S; start = initial bacterial community in shredded leaves, E; end = final bacterial community in shredded leaves, and F; bacterial community in faecal pellets. The two colors distinguish the samples from oxic or anoxic conditioning. The three different empty symbols indicate three leaf species, and the three different filled symbols indicate faecal pellets obtained from shredders after feeding those three leaf species.

For a more in‐depth evaluation of bacterial community turnover relative to the initial shredded leaves, we performed a PERMANOVA using Bray–Curtis dissimilarities of Hellinger-transformed 16S rRNA sequencing data and visualized it by NMDS. The structure of bacterial community composition differed across treatments, driven by leaf species, conditioning, and among shredded leaves versus faecal pellets (Fig. 3). The PERMANOVA results based on the abundances of bacterial taxa revealed a significant difference in bacterial communities across leaf species though the proportion of explained variation was relatively small (*F*_(2,35)_ = 5.51, *R*^2^ = 0.045, *P* < 0.001). In addition, we found a substantial turnover of bacterial communities from initial shredded leaves to those in final shredded leaves and faecal pellets (PERMANOVA, *F*_(2,35)_ = 50.61, *R*^2^ = 0.438, *P* < 0.001) and between oxic and anoxic conditioning (*F*_(1,35)_ = 52.96, *R*^2^ = 0.192, *P* < 0.001). Our NMDS plot showed the distinct clustering of bacterial communities based on conditioning and FPOM type. Bacterial communities of initial shredded leaves were markedly diverged from those in final shredded leaves and faecal pellets, along with oxic and anoxic conditioning forming well-separated clusters (Fig. 3).

### Indicator taxa of individual FPOM types

**Figure 4.**
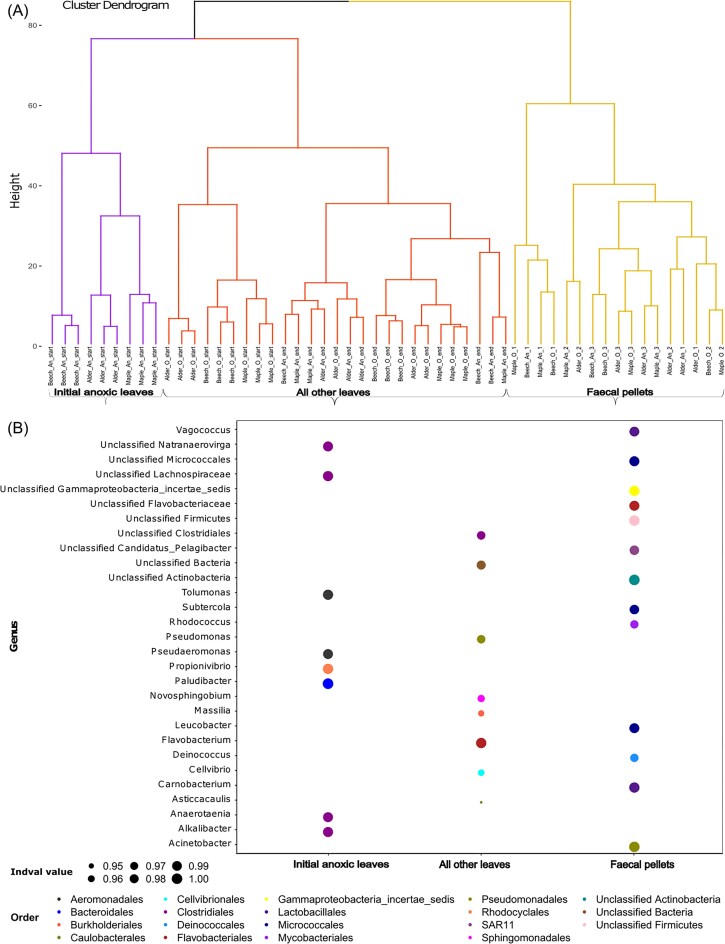
Representation of ISA. Hierarchical clustering (Ward’s method) of Euclidean distances among ASV relative abundances returned three clusters, representing initial anoxic shredded leaves (purple), all other shredded leaves, i.e. initial oxic and final shredded leaves (red), and faecal pellet samples (yellow) (A). Indicator taxa at order and genus-level for each group are shown below (B), in a bubble plot, where size of each circle represents the Indval value, which shows the association of each taxon to specific FPOM-type. Colors of each circle represent taxa at order level.

We performed a hierarchical clustering analysis on relative abundance of count taxon of ASVs, which showed the three clusters of taxa representing our initial anoxic shredded leaves, all other shredded leaves (oxic initial and the final shredded leaves), and faecal pellet samples (Fig. 4A). Our data showed that the bacterial taxa representing the initial anoxic shredded leaves were different from the other leaf communities including initial oxic shredded leaves and all final leaves. Indicator species analysis (ISA) on the relative abundance of the ASVs identified specialized, typical taxa associated with shredded leaves and faecal pellets and showed turnover in indicator taxa across time. Based on highest indval values obtained after ISA, the indicator taxa in initial anoxic leaves were *Alkalibacter, Anaerotaenia, Propionvibrio, Paludibacter*, and *Sulfurospirillum* (Table S10). *Asticcacaulis, Flavobacterium*, and *Pedobacter* were typical for all other shredded leaves and *Acinetobacter, Deinococcus, Carnobacterium, Subtercola, Rhodococcus*, and *Vagococcu*s were typical for faecal pellets (Fig. 4B; Table S10). Overall, *Paludibacter* had the highest Indval values (= 1) and thus was the most dominant taxon (*P* < 0.05) in initial anoxic leaves. Similarly, *Acinetobacter* and *Flavobacterium* were the most dominant taxa (*P* < 0.05) with indval value = 1 in faecal pellets and all other shredded leaves, respectively (Table S10).

## Discussion

In this study, we aimed to provide a new perspective on diversity and composition of bacterial communities associated with shredder-produced FPOM and thereby improve our understanding on the role of microbial communities associated with terrestrial-derived organic matter processing in streams. We found significant differences in diversity and taxonomic composition of bacterial communities associated with different fractions of shredder-produced FPOM but also revealed that properties of terrestrial organic matter (either intrinsic or due to microbial colonization) and passage through the shredders gut are important factors in shaping bacterial turnover, and reshaping the structure of bacterial communities associated to shredder-produced FPOM.

### Intrinsic leaf properties partially influence bacterial community composition

Our results show that the interaction between the leaf species and FPOM type had a significant effect on bacterial alpha diversity of shredder-produced FPOM (Table 1 and Table S9). We speculate that this difference can be attributed to the interplay between leaf properties and shredder activity since microbial colonization on leaves of varying C/N ratios may influence gut microbiota interaction and consequently influence FPOM attached microbial communities (Graça 2001, Wantzen and Wagner 2006, Villanueva et al. 2012, Ayayee et al. 2018, Gonçalves et al. 2024). Notably, we observed a higher bacterial diversity in alder compared to beech that was most prominent in the faecal pellets (Table S8). However, we did not find a strong leaf species effect on the Shannon diversity as well as the bacterial community composition (Fig. 3). Nevertheless, our results here align with prior findings, which emphasize that the initial leaf quality is a determining factor for the microbial assemblages (Graça 2001, Rezende et al. 2017). Alder, known for its higher nutrient content and lower structural complexity (e.g. lignin concentration), appears to provide a more favorable environment for microbial colonization compared to low-quality leaves such as beech (Hladyz et al. 2009, Kominoski et al. 2009). In addition, we assume that the selective feeding of shredders on certain parts of the leaves (Fenoy et al. 2021), such as the veins versus the softer tissue, played a crucial role in modifying the leaf properties which in turn, may have resulted in the processing of leaf components of differing toughness differently. This might have sped-up or slowed down the gut passage for the ingested material, altering the substrate quality, surface area, or gut passage time for microbial colonization, thereby modulating microbial diversity in faecal pellets (Bärlocher and Kendrick 1975, Hieber and Gessner 2002, Yoshimura et al. 2008, Cheever et al. 2013). Hence, we here speculate that the combined effect of alder’s intrinsic chemical properties and shredder-mediated processing may have contributed to higher bacterial diversity in alder-derived FPOM and potentially increasing the LLD rate and efficiency in streams.

Moreover, we found that, over time, many ASVs that were abundant in initial shredded leaves, such as Gammaproteobacteria decreased in abundance while other ASVs such as Betaproteobacteria increased (Fig. 1) in all leaf treatments as described in other stream ecosystem studies (Newman et al. 2015, Zhao et al. 2017). Over time, alpha diversity and abundance of bacterial communities increased under aerobic conditions by following species–time relationships patterns as shown in other ecosystems (Shade et al. 2013, Karačić et al. 2022). As the biofilm matures and bacteria metabolize the cell polymers and excrete metabolites creating broader environmental niches (Karačić et al. 2024). This possibly resulted in the establishment of more diverse biofilm communities and consequently increasing diversity in final shredded leaves (Tláskal et al. 2016, Zhao et al. 2017). It is likely that more diverse communities compete or coexist within available resources and may have several metabolic functions such as carbon and nitrogen metabolism and nutrient release, potentially making a stream ecosystem resilient under different disturbances (Ramond et al. 2024). Furthermore, the increasing predominance of Betaproteobacteria in our experiments resembles bacterial communities in biofilms and surface water samples (Araya et al. 2003) and suggests that our experiments likely captured near-natural community turnover dynamics, where changes in the chemical properties of leaf litter are reflected in succession of communities (Raposeiro et al. 2017). In addition, introducing shredder larvae may have simply resulted in an inoculation of the water in the microcosms by gut-associated bacteria that differed from the original stream water community. The changes in light conditions during the experiment (from dark during conditioning to 11:13 h light: dark cycles) may have further resulted in algal growth, thereby increasing the resource pool for heterotrophic bacteria through exudate production (Wyatt and Turetsky 2015, Francoeur et al. 2020, Pope et al. 2020). Altogether, we argue that changes in leaf chemical composition as well as the activity of the experimental macroinvertebrate shredders fostered community turnover and resulted in an overall higher diversity of bacterial communities in final leaves compared to initial leaves.

In contrast, the differences observed in bacterial communities among faecal pellets produced by feeding different leaf species, as compared to those found on shredded leaves (Figs 1 and 2B) may indicate that the shredders’ gut selectively modifies bacterial assemblages based on the leaf properties. However, other factors such as macroinvertebrate feeding preferences and gut conditions (i.e. pH and oxygen level) may have mediated microbial communities and microbial growth efficiency could play a role in LLD (Danger et al. 2012, Foucreau et al. 2013). Our finding thus points out the differences in digestion processes during consumption of varying leaves with different qualities, suggesting the shredders’ gut may selectively boost or suppress the taxa based on the nutrient content and degradability of the ingested leaves (Foucreau et al. 2013, Halvorson et al. 2014). This selection process during gut passage likely enhances both taxonomic and functional diversity of the stream microbiome and thereby may strongly influence organic matter remineralization, storage, and production.

### Conditioning affects bacterial community composition

Leaf conditioning under oxic or anoxic conditions prior to the feeding experiment had a substantial impact on bacterial diversity and composition in our experiments: leaves conditioned in an oxic environment, along with the experimental leaves from the final sampling at the end of the incubation, exhibited greater bacterial diversity. They were dominated by aerobic taxa such as *Cellvibrio* (Order: Cellvibrionales), *Flavobacterium* (Flavobacteriales), *Sphingomonas* (Sphingomonadales), *Pseudomonas* (Pseudomonadales), and *Massilia* (Burkholderiales). These genera are commonly known leaf litter decomposers and have the ability to degrade plant-derived polymers (Rastogi et al. 2012, Tláskal et al. 2016, Silverman et al. 2017, Janssen et al. 2021, Khomutovska et al. 2024). Such aerobic and facultative aerobic taxa use oxidative metabolic pathways to break down complex organic compounds, such as lignin and cellulose (Gessner and Chauvet 1994, Ma et al. 2020). This potentially accelerates the production of FPOM by invertebrate shredders (Baldy et al. 2002, Gessner 2010, Danger et al. 2012). Indeed, we found several ASVs on the oxic-conditioned leaves corroborating this interpretation. For instance, we found *Sphingomonas* that is able to produce and use *N*-acetyl-d-glucosamine, a monomer of the fungal polymer chitin (Valášková et al. 2009) while *Pedobacter* and *Pseudomonas* have been previously well-described for chitinolytic activity (Brabcová et al. 2016), which may direct towards a fungi–bacteria interplay during LLD. In addition, *Sulfurospirillum*, another genus of bacterial leaf decomposers (Vesamäki et al. 2024), was found. The occurrences of these taxa suggest that they can utilize leaf litter as their energy source and assist the leaf breakdown process rapidly.

In contrast, ASVs assigned to the genera *Propionivibrio* (Order; Rhodocylales), *Alkalibacter* (Clostridiales), *Paludibacter* (Bacteroidales), *Pseudaeromonas* (Gammaproteobacteria), *Tolumonus* (Gammaproteobacteria), and others were identified as indicative taxa, dominating the initial shredded leaves under anoxic conditions. These taxa are adapted to low-oxygen environments (Karačić et al. 2024), and are known for their role in anaerobic metabolism and early colonization of organic substrates (Hau and Gralnick 2007, Smeaton et al. 2009, Qiu et al. 2017, Silverman et al. 2017). We found the genus *Shewanella* on initial shredded leaves under anoxic conditions, which is known as iron-reducer and also transforms the heavy metals, such as lead and chromium into less or nontoxic forms through anaerobic respiration pathways (Hau and Gralnick 2007, Smeaton et al. 2009). Rhodocyclales, another group of versatile bacteria, are known for utilizing a wide variety of organic compounds for growth (Kittichotirat et al. 2011). These bacterial taxa, using mostly anaerobic metabolic pathways, such as fermentation, decompose organic matter somewhat slower (Degelmann et al. 2009, Qiu et al. 2017, Huang et al. 2022), yet may contribute to a pool of fermentative products upon reexposure to oxygen. Overall, our findings on the bacterial communities on initial CPOM align with previous results on the role of oxygen availability in shaping microbial decomposition strategies (Huang et al. 2022). Interestingly, the communities established under oxic and anoxic conditions persisted to some degree throughout leaf particle passage through shredders guts, suggesting that the environmental history continues to influence the microbial succession trajectories, even after gut passage.

### Gut passage effects: input is not the same as output

Faecal pellets, one type of FPOM, differed substantially in their bacterial communities compared to initial CPOM and final shredded leaves, independent of the leaf species as supported by an overall shift in community structure and the ISA. These shifts in community composition from leaves to faecal pellets are possibly due to selective digestion, specific gut environments, such as pH levels, oxygen gradients, or the introduction of gut-associated bacteria (Danger et al. 2012, Wei et al. 2014, Jung and Park 2015, Ayayee et al. 2018). In addition, ISA revealed that certain taxa were found solely in faecal pellets, suggesting that gut passage plays a crucial role in shaping the FPOM attached bacterial community composition.

Particularly, anaerobic or facultative anaerobic bacterial taxa became dominant on faecal pellets while those less adapted to gut conditions were reduced or digested in the shredders’ gut. Our findings thus resemble previous results on faecal pellets of other detritivores, i.e. earthworms which showed a reduced bacterial alpha diversity after gut passage compared to the ingested soil (Aira et al. 2022) due to the homogenization of the bacterial community and the loss of rare taxa (Plante et al. 1996, Graça 2001, Yoshimura et al. 2008). Indeed, gut passage promotes the assembly of unique communities on faecal pellets suggesting that invertebrate taxa can modify the microbial community composition during gut passage, presumably, via selective digestion or preferential reduction of specific taxa (Plante et al. 1996, Graça 2001).

Our analysis also revealed a notable shift in the relative abundance of bacterial taxa, a clear decrease in Bacteroidetes and an increase in Firmicutes and Proteobacteria in the faecal pellets compared to the initial shredded leaves (Fig. S1; Table S7). Bacteroidetes are well-studied abundant surface colonizers of particulate organic matter and play a key role in their degradation and utilization (Crump et al. 1999, Riemann and Winding 2001, Kirchman 2002). Reduction of these Bacteroidetes taxa on faecal pellet may reflect a shift in metabolic pathways of communities reshaped by gut passage. Notably, Pseudomonadales (∼24%) was the most frequently detected proteobacterial order in faecal pellet samples, followed by Burkholderiales (∼12%) and Enterobacteriales (∼10%), all of which can degrade a vast array of long-chain dicarboxylic acids and hydroxylated aromatic compounds including lignin and constituents of plant exudates (Pérez-Pantoja et al. 2012, Ghosal et al. 2016). This selective turnover and enrichment likely reflect the role of the shredders’ gut as a microbial filter and bioreactor, altering bacterial diversity and community composition through selective retention or enhancement during the digestion process. Further, the faecal pellets were enriched in taxa such as *Acinetobacter, Carnobacterium*, and *Rhodococcus*, which are known for their ability to metabolize complex organic compounds via fermentation and survive under low-nutrient conditions (Leisner et al. 2007, Jung and Park 2015). In addition, we observed ASVs belonging to the genera *Carnobacterium* (Class: Bacilli), *Leucobacter* (Actinobacteria), *Acinetobacter* (Gammaproteobacteria), *Vagococcus* (Bacilli), and *Rhodococcus* (Actinobacteria) as indicator species in faecal pellets (Table S10), which are commonly reported as gut bacteria of Diptera larvae (Sontowski and van Dam 2020) and other detritivores such as silkworm (Gogoi et al. 2023) and green bottle fly larvae. It is likely that these taxa colonized the particles in the shredder’s gut and may contribute to further processing of the egested particles.

To date, there are some studies reporting gut microbiomes of different functional feeding groups (filter feeders, grazers/collectors, predators, and omnivores) of benthic invertebrates in stream ecosystems (Ayayee et al. 2018, 2022). Yet, information on the gut microbiome of shredders, especially in the order *Trichoptera*, which is one of the most important functional feeding groups in headwater streams, is still missing. Our study is the first, to address the transition in structure and taxonomic composition of bacterial communities during the conversion of leaves into faecal pellets by feeding activity of *Sericostoma* sp (Insecta: Trichoptera). Hence, these findings suggest that shredders not only contribute to the physical breakdown of leaves but also change the microbial community composition on the shredder-derived FPOM via the digestion process, thereby probably facilitating the microbial conversion of organic matter into simpler forms, which can be utilized by downstream consumers such as filter-feeders.

The prevalence of different bacterial taxonomic groups (phylum, class, and genera) in shredded leaves compared to faecal pellets revealed that the bacterial communities colonizing the leaves are not similar to the communities colonizing faecal pellets during LLD in streams. In the light of this observation, we may presume that the combination of environmental factors e.g. oxygen levels, and gut passage, which either may have digested several bacterial taxa or favored the growth of individual bacterial taxa or introduced gut residents in faecal pellets shaped the microbial communities in each FPOM type. Our data thus suggest that the bacterial assemblages on different FPOM types may employ various metabolic pathways, which possibly contribute diversely to LLD in streams with potential implications for downstream nutrient cycling, and food web dynamics.

### Limitations and future directions of the study

While our microcosm study provides valuable insights related to LLD in streams, it oversimplified the complexity of natural stream ecosystems. For instance, we focused on the importance of leaf species in shaping the bacterial community composition associated with shredder-produced FPOM, where our interpretations are limited by the number of leaf species selected. Including a broader range of leaf types with a greater range of properties (e.g. evergreen versus deciduous leaves and native versus invasive leaves) could provide better insights into how CPOM substrates affect FPOM-associated microbial communities and their role in LLD in streams. Moreover, the complexities of natural stream ecosystems regarding oxygen conditions and other environmental characteristics, such as variation in flow, organic load, or temperatures, but also interactions with other microbes such as algae and fungi may modulate bacterial communities even further. These may influence diversity and composition patterns of bacterial communities associated with CPOM and shredder-produced FPOM to a much greater degree with important implications for the stability and resilience of ecosystems. Moreover, integrating fungal community dynamics, which are critical during the initial stage of LLD, and examining bacterial communities inhabiting the gut of freshwater shredders across shredder species and developmental stages could further explain the complex interactions between shredders, their food resources, and microbial dynamics. Lastly, it is crucial to link functional capacities of microbial composition associated with FPOM, such as enzyme activities, metabolism, and nutrient fluxes. All these factors will be essential to fully understand the role of microbial assemblages in stream ecosystem functioning.

## Conclusion

Bacterial communities dwelling on the FPOM produced during LLD can play a pivotal role for energy flow and nutrient cycling within stream food webs. Yet, the interactions between different leaf species and their microbial assemblages under different environmental conditions, as well as how these interactions determine the shredder activity and consequently, the bacterial assembly in the shredder-produced FPOM are not yet well understood. Our study sheds light on these aspects. We found that oxic conditioning supported higher bacterial diversity, potentially enhancing terrestrial organic matter processing in streams. We also observed that the interplay between the leaf species and shredder activity was a determinant of bacterial diversity across different FPOM types. Nevertheless, we found no clear evidence of distinct differences in alpha diversity or community composition between the three leaf species, which necessitates more targeted research on this topic. Importantly, faecal pellets exhibited distinct bacterial communities compared to that of leaf substrates, indicating that gut passage drives turnover and reshapes bacterial communities on fine particles in streams. In addition, we showed that specific bacterial communities dominated each FPOM type and discussed their potential significance during LLD. Such bacterial communities colonizing the FPOM subsequently could determine the fate of shredder-produced FPOM in streams. The variability in FPOM associated bacterial communities we detected in our study highlights how biotic interactions can shape the microbial diversity and ecosystem processes in streams. Understanding these dynamics may assist to predict how stream ecosystems respond to several environmental changes such as altered riparian inputs, oxygen dynamics, or biodiversity loss.

## Supplementary Material

fiaf091_Supplemental_File

## Data Availability

The 16S rRNA gene sequencing data generated in this study have been deposited in the GenBank database under the BioProject ID_PRJNA1258879. All other relevant data supporting the findings of this study are available within the article and its supplementary information.
