# Supplementary material for: Distinct bacterial communities affiliated with two types of shredder-produced particles in streams
Source: FEMS Microbiol Ecol. 2025 Sep 16;101(10):fiaf091. doi: 10.1093/femsec/fiaf091 (PMC12451447; doi:10.1093/femsec/fiaf091)
Supplement: fiaf091_Supplemental_File [file fiaf091_supplemental_file.docx]

Title: Distinct bacterial communities affiliated with two types of shredder-produced particles in streams

Pratiksha Acharya^1,2*^, Mourine J. Yegon^1,3^, Christian Griebler^2^, Simon Vitecek^3,4#^, Katrin Attermeyer^1,2#^

^1^ WasserCluster Lunz – Biological Station, Dr. Carl Kupelwieser-Prom. 5, 3293 Lunz am See, Austria

^2^ Department of Functional and Evolutionary Ecology, Unit Limnology, University of Vienna, Djerassiplatz 1, 1030 Vienna, Austria

^3^ Institute for Hydrobiology and Water Management (IHG), University of Natural Resources and Life Sciences, Gregor-Mendel-Straße 33/DG, 1180 Vienna, Austria

^4^ Department of Ecology, University of Innsbruck, Technikerstraße 25, 6020 Innsbruck, Austria

*^*^ Correspondence:* Pratiksha.Acharya@wcl.ac.at

# Shared senior authors


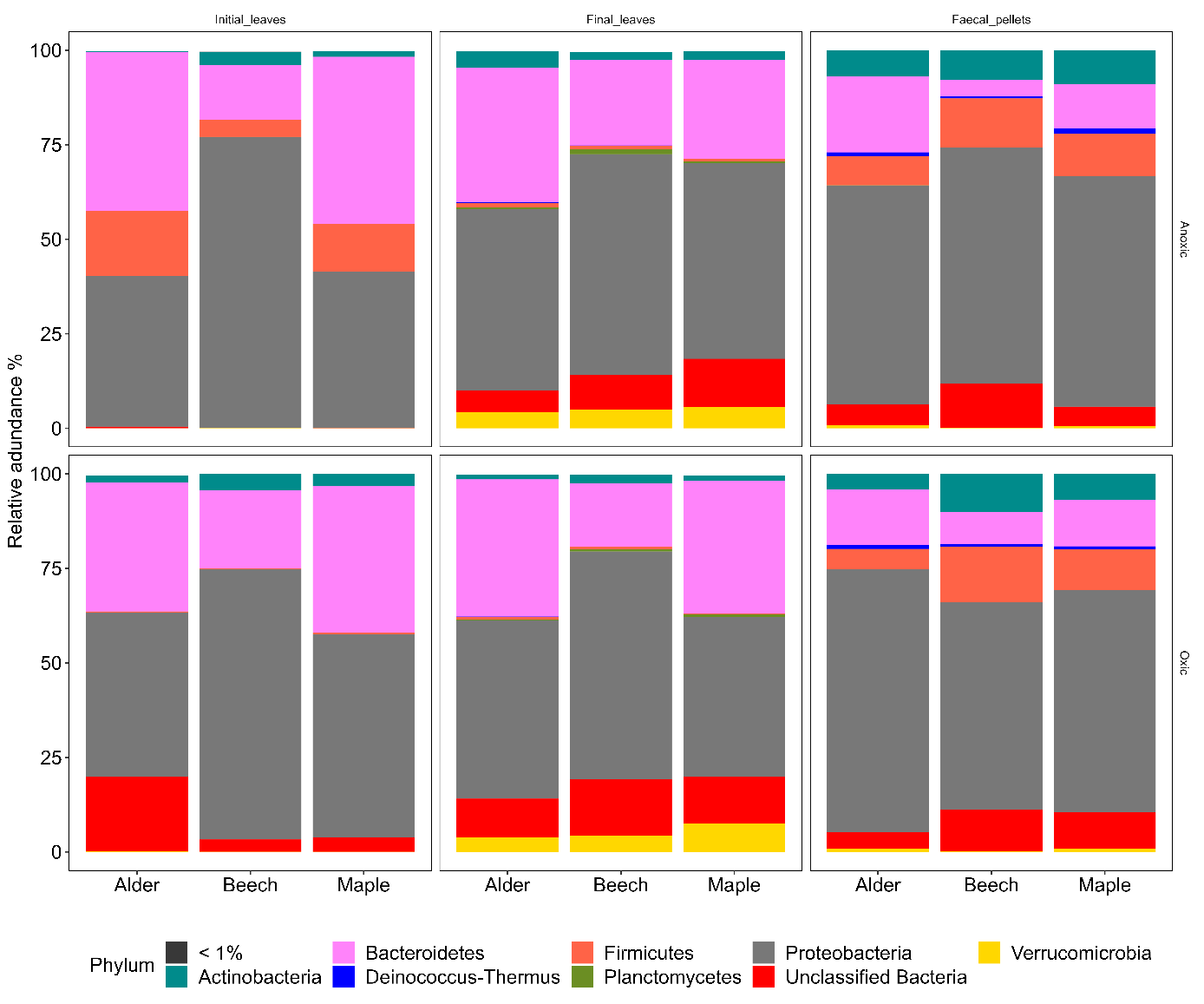


Fig. S1. Relative abundance of ASVs belonging to different FPOM types at a phylum level are shown. ASVs that were present in less than 1 % were grouped in one as “< 1 %”.

**Table. S1.** Differences in bacterial phylum level relative abundance among FPOM types i.e., initial leaves, final leaves and faecal pellets. A Kruskal-Wallis test with a Benjamini-Hochberg (BH) correction was used to compare among the three groups. Only bacterial phyla with greater than 1% of the total relative abundance across all samples are shown. Abundances are given as mean ± SE. Abbreviations; p: p-values, p.adj: p-adjusted values.

| **Phylum** | **p** | **p.adj** | **Initial leaves Abundance** | **Final leaves Abundance** | **Faecal pellets Abundance** |
| --- | --- | --- | --- | --- | --- |
|  |  |  | **(N=18)** | **(N=18)** | **(N=17)** |
| Actinobacteria | **0.006** | **0.011** | 2.46±0.37 | 2.21±0.39 | 7.28±1.03 |
| Bacteroidetes | **0.008** | **0.012** | 32.32±2.81 | 28.782±2.21 | 12.12±1.67 |
| Deinococcus-Thermus | **0.002** | **0.006** | 0.015±0.004 | 0.069±0.02 | 0.95±0.23 |
| Firmicutes | **0.042** | 0.05 | 5.87±1.66 | 0.69±0.09 | 10.14±1.11 |
| Planctomycetes | **<0.001** | **0.003** | 0.004±0.002 | 0.65±0.11 | 0.05±0.007 |
| Proteobacteria | 0.198 | 0.200 | 54.46±3.71 | 51.26±2.42 | 61.11±3.03 |
| Unclassified Bacteria | **0.045** | 0.05 | 4.58±1.72 | 10.93±1.29 | 7.68±2.47 |
| Verrucomicrobia | **<0.001** | **0.003** | 0.03±0.007 | 5.03±0.48 | 0.59±0.17 |

***Statistically significant p-values (< 0.05) values are shown in bold***

**Table. S2.** Differences in bacterial class level relative abundance among FPOM types i.e., initial leaves, final leaves and faecal pellets. A Kruskal-Wallis test with a Benjamini-Hochberg (BH) correction was used to compare among the three groups. Only bacterial class with greater than 1% of the total relative abundance across all samples are shown. Abundances are given as mean ± SE. Abbreviations; p: p-values, p.adj: p-adjusted values.

| **Class** | **p** | **p.adj** | **Initial leaves**  **Abundance**  **(N=18)** | **Final leaves**  **Abundance**  **(N=18)** | **Faecal pellets Abundance**  **(N=17)** |
| --- | --- | --- | --- | --- | --- |
| Actinobacteria | **0.006** | **0.009** | 2.45±0.37 | 2.13±0.38 | 7.22±1.03 |
| Alphaproteobacteria | 0.691 | 0.730 | 12.90±1.48 | 13.46±0.99 | 11.49±1.78 |
| Bacilli | **0.003** | **0.006** | 0.12±0.03 | 0.064±0.02 | 9.09±1.05 |
| Bacteroidia | 0.777 | 0.780 | 9.38±2.86 | 0.93±0.23 | 0.22±0.04 |
| Betaproteobacteria | **0.006** | **0.009** | 12.02±0.98 | 24.39±1.39 | 13.80±1.32 |
| Chitinophagia | **<0.001** | **0.002** | 0.007±0.002 | 0.91±0.08 | 0.22±0.05 |
| Clostridia | 0.117 | 0.150 | 5.611±1.61 | 0.60±0.08 | 0.27±0.08 |
| Cytophagia | 0.281 | 0.330 | 5.56±1.55 | 7.84±0.59 | 4.62±1.01 |
| Deinococci | **0.002** | **0.005** | 0.015±0.003 | 0.07±0.023 | 0.96±0.23 |
| Flavobacteriia | **0.015** | **0.021** | 15.29±1.42 | 15.46±1.79 | 6.34±0.87 |
| Gammaproteobacteria | **0.004** | **0.006** | 29.51±3.65 | 12.37±1.12 | 35.53±2.43 |
| Opitutae | **<0.001** | **0.0015** | 0.004±0.001 | 0.85±0.16 | 0.12±0.03 |
| Planctomycetacia | **<0.001** | **0.0015** | 0.004±0.002 | 0.65±0.11 | 0.05±0.01 |
| Sphingobacteriia | 0.324 | 0.360 | 2.07±0.52 | 0.72±0.21 | 0.33±0.23 |
| Unclassified Bacteria | **0.045** | 0.059 | 4.58±1.73 | 10.93±1.29 | 7.68±2.48 |
| Verrucomicrobiae | **<0.001** | **0.0015** | 0.005±0.001 | 3.79±0.37 | 0.42±0.13 |

***Statistically significant p-values (< 0.05) values are shown in bold***

**Table. S3.** Differences in bacterial phylum level relative abundance between conditioning i.e., anoxic and oxic. A Kruskal-Wallis test with a Benjamini-Hochberg (BH) correction was used to compare between the two groups. Only bacterial phyla with greater than 1% of the total relative abundance across all samples are shown. Abundances are given as mean ± SE. Abbreviations; p: p-values, p.adj: p-adjusted values.

| **Phylum** | **p** | **p.adj** | **Anoxic**  **Abundance**  **(N=27)** | | **Oxic**  **Abundance**  **(N=26)** |
| --- | --- | --- | --- | --- | --- |
| Actinobacteria | 0.825 | 0.89 | | 4.15±0.73 | 3.69±0.65 |
| Bacteroidetes | 0.895 | 0.89 | | 24.59±2.73 | 24.70±2.31 |
| Deinococcus-Thermus | 0.690 | 0.89 | | 0.35±0.13 | 0.32±0.14 |
| Firmicutes | 0.058 | 0.52 | | 7.63±1.20 | 3.24±1.04 |
| Planctomycetes | 0.757 | 0.89 | | 0.29±0.10 | 0.19±0.05 |
| Proteobacteria | 0.895 | 0.89 | | 55.28±2.81 | 55.74±2.43 |
| Unclassified Bacteria | 0.171 | 0.77 | | 5.65±1.57 | 9.89±1.51 |
| Verrucomicrobia | 0.566 | 0.89 | | 1.80±0.49 | 2.02±0.52 |

**Table. S4.** Differences in bacterial phylum level relative abundance among treatment i.e., alder, beech and maple. A Kruskal-Wallis test with a Benjamini-Hochberg (BH) correction was used to compare among the three groups. Only bacterial phyla with greater than 1% of the total relative abundance across all samples are shown. Abundances are given as mean ± SE. Abbreviations; p: p-values, p.adj: p-adjusted values

| **Phylum** | **p** | **p.adj** | **Alder**  **Abundance**  **(N=18)** | **Beech**  **Abundance**  **(N=17)** | **Maple**  **Abundance**  **(N=18)** |
| --- | --- | --- | --- | --- | --- |
| Actinobacteria | 0.385 | 0.98 | 3.07±0.75 | 4.73±0.95 | 4.01±0.84 |
| Bacteroidetes | 0.091 | 0.41 | 30.45±2.54 | 14.87±1.99 | 28.06±3.24 |
| Deinococcus-Thermus | 0.927 | 0.98 | 0.41±0.19 | 0.19±0.10 | 0.39±0.18 |
| Firmicutes | 0.960 | 0.98 | 5.39±1.45 | 5.11±1.46 | 5.91±1.55 |
| Planctomycetes | 0.894 | 0.98 | 0.15±0.05 | 0.36±0.14 | 0.21±0.07 |
| Proteobacteria | 0.050 | 0.41 | 50.97±2.72 | 64.57±3.10 | 51.47±2.82 |
| Unclassified Bacteria | 0.983 | 0.98 | 7.66±1.68 | 8.24±2.52 | 7.33±1.66 |
| Verrucomicrobia | 0.884 | 0.98 | 1.63±0.47 | 1.66±0.55 | 2.41±0.79 |

**Table. S5.** Differences in bacterial class level relative abundance among treatment i.e., alder, beech and maple. A Kruskal-Wallis test with a Benjamini-Hochberg (BH) correction was used to compare among the three groups. Only bacterial class with greater than 1% of the total relative abundance across all samples are shown. Abundances are given as mean ± SE. Abbreviations; p: p-values, p.adj: p-adjusted values.

| **Class** | **p** | **p.adj** | **Alder**  **Abundance**  **(N=18)** | **Beech**  **Abundance**  **(N=17)** | **Maple**  **Abundance**  **(N=18)** |
| --- | --- | --- | --- | --- | --- |
| Actinobacteria | 0.444 | 0.99 | 3.04±0.74 | 4.67±0.95 | 3.96±0.84 |
| Alphaproteobacteria | 0.368 | 0.99 | 11.07±1.09 | 14.83±1.67 | 12.15±1.43 |
| Bacilli | 0.884 | 0.99 | 1.90±0.64 | 3.66±1.41 | 3.42±1.32 |
| Bacteroidia | 0.423 | 0.99 | 5.45±2.55 | 1.19±0.51 | 3.96±2.01 |
| Betaproteobacteria | 0.529 | 0.99 | 18.21±1.09 | 16.61±2.28 | 15.55±1.92 |
| Chitinophagia | 0.834 | 0.99 | 0.30±0.07 | 0.41±0.13 | 0.43±0.12 |
| Clostridia | 0.692 | 0.99 | 3.17±1.45 | 1.05±0.41 | 2.30±1.11 |
| Cytophagia | 0.331 | 0.99 | 7.03±1.04 | 3.97±0.88 | 6.99±1.36 |
| Deinococci | 0.927 | 0.99 | 0.41±0.18 | 0.19±0.09 | 0.39±0.18 |
| Flavobacteriia | 0.069 | 0.99 | 15.77±1.71 | 7.11±0.97 | 14.25±1.68 |
| Gammaproteobacteria | 0.421 | 0.99 | 21.28±2.54 | 32.75±4.37 | 23.21±2.97 |
| Opitutae | 0.805 | 0.99 | 0.21±0.05 | 0.26±0.09 | 0.51±0.19 |
| Planctomycetacia | 0.894 | 0.99 | 0.15±0.04 | 0.36±0.15 | 0.21±0.07 |
| Saprospiria | 0.994 | 0.99 | 0.21±0.05 | 0.37±0.12 | 0.36±0.12 |
| Sphingobacteriia | 0.587 | 0.99 | 1.26±0.42 | 0.65±0.29 | 1.23±0.43 |
| Unclassified Bacteria | 0.983 | 0.99 | 7.66±1.68 | 8.24±2.52 | 7.33±1.66 |
| Unclassified Bacteroidetes | 0.982 | 0.99 | 0.43±0.11 | 1.17±0.41 | 0.84±0.26 |
| Unclassified Firmicutes | 0.729 | 0.99 | 0.21±0.09 | 0.39±0.16 | 0.18±0.07 |
| Verrucomicrobiae | 0.750 | 0.99 | 1.34±0.41 | 1.15±0.39 | 1.77±0.59 |

**Table. S6.** Differences in bacterial class level relative abundance between conditioning i.e., anoxic and oxic. A Kruskal-Wallis test with a Benjamini-Hochberg (BH) correction was used to compare between the two groups. Only bacterial class with greater than 1% of the total relative abundance across all samples are shown. Abundances are given as mean ± SE. Abbreviations; p: p-values, p.adj: p-adjusted values.

| **Class** | **p** | **p.adj** | **Anoxic**  **Abundance**  **(N=27)** | **Oxic**  **Abundance**  **(N=26)** |
| --- | --- | --- | --- | --- |
| Actinobacteria | 0.895 | 0.94 | 4.09±0.73 | 3.65±0.65 |
| Alphaproteobacteria | **0.002** | **0.017** | 9.95±0.99 | 15.43±1.09 |
| Bacilli | 0.310 | 0.89 | 3.17±0.92 | 2.78±0.99 |
| Bacteroidia | **0.009** | **0.046** | 6.77±2.03 | 0.25±0.11 |
| Betaproteobacteria | 0.691 | 0.94 | 17.20±1.63 | 16.38±1.31 |
| Chitinophagia | 0.825 | 0.94 | 0.37±0.09 | 0.39±0.09 |
| Clostridia | **0.005** | **0.036** | 4.08±1.14 | 0.24±0.05 |
| Cytophagia | 0.171 | 0.57 | 4.32±0.88 | 7.81±0.89 |
| Deinococci | 0.690 | 0.94 | 0.35±0.13 | 0.32±0.14 |
| Flavobacteriia | 0.965 | 0.96 | 12.08±1.41 | 12.89±1.46 |
| Gammaproteobacteria | 0.566 | 0.94 | 27.72±3.05 | 23.44±2.61 |
| Opitutae | 0.690 | 0.94 | 0.31±0.12 | 0.36±0.09 |
| Planctomycetacia | 0.757 | 0.94 | 0.29±0.10 | 0.19±0.05 |
| Saprospiria | 0.787 | 0.94 | 0.34±0.09 | 0.29±0.07 |
| Sphingobacteriia | **0.001** | **0.017** | 0.07±0.02 | 2.08±0.37 |
| Unclassified Bacteria | 0.171 | 0.57 | 5.65±1.57 | 9.89±1.51 |
| Unclassified Bacteroidetes | 0.688 | 0.94 | 0.63±0.17 | 0.99±0.28 |
| Unclassified Firmicutes | 0.857 | 0.94 | 0.30±0.08 | 0.22±0.11 |
| Verrucomicrobiae | 0.626 | 0.94 | 1.36±0.37 | 1.49±0.40 |

***Statistically significant p-values (< 0.05) values are shown in bold***

**Table S7.** Taxonomic classification of bacterial communities with more than 5 % relative abundances present in different treatments are shown here.

| **Sample_type** | **Leaf species** | **Conditioning** | **Phylum** | **Class** | **Order** | **Family** | **Genus** | **Relative abundance [%]** |
| --- | --- | --- | --- | --- | --- | --- | --- | --- |
| Initial_leaves | Alder | Anoxic | Bacteroidetes | Bacteroidia | Bacteroidales | Paludibacteraceae | Paludibacter | 27.585 |
|  | Alder | Oxic | Bacteroidetes | Flavobacteriia | Flavobacteriales | Flavobacteriaceae | Flavobacterium | 21.487 |
|  | Alder | Oxic | Unclassified Bacteria | Unclassified Bacteria | Unclassified Bacteria | Unclassified Bacteria | Unclassified Bacteria | 19.791 |
|  | Alder | Anoxic | Bacteroidetes | Flavobacteriia | Flavobacteriales | Flavobacteriaceae | Flavobacterium | 13.236 |
|  | Alder | Anoxic | Proteobacteria | Betaproteobacteria | Rhodocyclales | Zoogloeaceae | Uliginosibacterium | 12.469 |
|  | Alder | Oxic | Proteobacteria | Gammaproteobacteria | Pseudomonadales | Pseudomonadaceae | Pseudomonas | 10.363 |
|  | Alder | Anoxic | Firmicutes | Clostridia | Clostridiales | Natranaerovirga | Unclassified Natranaerovirga | 5.663 |
|  | Alder | Anoxic | Firmicutes | Clostridia | Clostridiales | Lachnospiraceae | Unclassified Lachnospiraceae | 5.428 |
|  | Alder | Anoxic | Proteobacteria | Gammaproteobacteria | Aeromonadales | Aeromonadaceae | Aeromonas | 5.055 |
|  | Beech | Anoxic | Proteobacteria | Gammaproteobacteria | Aeromonadales | Aeromonadaceae | Aeromonas | 22.701 |
|  | Beech | Oxic | Proteobacteria | Gammaproteobacteria | Pseudomonadales | Pseudomonadaceae | Pseudomonas | 22.341 |
|  | Beech | Anoxic | Proteobacteria | Gammaproteobacteria | Aeromonadales | Aeromonadaceae | Tolumonas | 18.507 |
|  | Beech | Oxic | Bacteroidetes | Flavobacteriia | Flavobacteriales | Flavobacteriaceae | Flavobacterium | 8.907 |
|  | Beech | Anoxic | Bacteroidetes | Flavobacteriia | Flavobacteriales | Flavobacteriaceae | Flavobacterium | 8.849 |
|  | Beech | Anoxic | Proteobacteria | Gammaproteobacteria | Pseudomonadales | Pseudomonadaceae | Pseudomonas | 7.852 |
|  | Beech | Oxic | Proteobacteria | Gammaproteobacteria | Enterobacterales | Erwiniaceae | Pantoea | 7.316 |
|  | Beech | Oxic | Bacteroidetes | Cytophagia | Cytophagales | Cytophagaceae | Arcicella | 6.423 |
|  | Beech | Oxic | Proteobacteria | Alphaproteobacteria | Sphingomonadales | Sphingomonadaceae | Sphingomonas | 6.127 |
|  | Beech | Anoxic | Proteobacteria | Gammaproteobacteria | Enterobacterales | Enterobacteriaceae | Klebsiella | 5.320 |
|  | Maple | Anoxic | Bacteroidetes | Flavobacteriia | Flavobacteriales | Flavobacteriaceae | Flavobacterium | 21.242 |
|  | Maple | Oxic | Bacteroidetes | Flavobacteriia | Flavobacteriales | Flavobacteriaceae | Flavobacterium | 16.539 |
|  | Maple | Anoxic | Bacteroidetes | Bacteroidia | Bacteroidales | Paludibacteraceae | Paludibacter | 16.203 |
|  | Maple | Anoxic | Proteobacteria | Gammaproteobacteria | Aeromonadales | Aeromonadaceae | Tolumonas | 14.602 |
|  | Maple | Oxic | Proteobacteria | Gammaproteobacteria | Pseudomonadales | Pseudomonadaceae | Pseudomonas | 12.805 |
|  | Maple | Oxic | Bacteroidetes | Cytophagia | Cytophagales | Cytophagaceae | Arcicella | 9.751 |
|  | Maple | Anoxic | Firmicutes | Clostridia | Clostridiales | Lachnospiraceae | Unclassified Lachnospiraceae | 6.116 |
|  | Maple | Anoxic | Bacteroidetes | Bacteroidia | Bacteroidales | Marinilabiliaceae | Breznakibacter | 5.548 |
|  | Maple | Anoxic | Proteobacteria | Betaproteobacteria | Rhodocyclales | Zoogloeaceae | Uliginosibacterium | 5.107 |
|  | Maple | Anoxic | Proteobacteria | Gammaproteobacteria | Aeromonadales | Aeromonadaceae | Aeromonas | 5.003 |
| Final_leaves | Alder | Oxic | Bacteroidetes | Flavobacteriia | Flavobacteriales | Flavobacteriaceae | Flavobacterium | 22.468 |
|  | Alder | Anoxic | Bacteroidetes | Flavobacteriia | Flavobacteriales | Flavobacteriaceae | Flavobacterium | 21.634 |
|  | Alder | Oxic | Unclassified Bacteria | Unclassified Bacteria | Unclassified Bacteria | Unclassified Bacteria | Unclassified Bacteria | 10.225 |
|  | Alder | Anoxic | Unclassified Bacteria | Unclassified Bacteria | Unclassified Bacteria | Unclassified Bacteria | Unclassified Bacteria | 5.790 |
|  | Alder | Anoxic | Proteobacteria | Gammaproteobacteria | Pseudomonadales | Moraxellaceae | Acinetobacter | 5.739 |
|  | Alder | Oxic | Proteobacteria | Betaproteobacteria | Burkholderiales | Comamonadaceae | Unclassified Comamonadaceae | 5.051 |
|  | Beech | Oxic | Unclassified Bacteria | Unclassified Bacteria | Unclassified Bacteria | Unclassified Bacteria | Unclassified Bacteria | 14.972 |
|  | Beech | Anoxic | Proteobacteria | Gammaproteobacteria | Pseudomonadales | Moraxellaceae | Acinetobacter | 12.234 |
|  | Beech | Anoxic | Bacteroidetes | Flavobacteriia | Flavobacteriales | Flavobacteriaceae | Flavobacterium | 9.974 |
|  | Beech | Anoxic | Unclassified Bacteria | Unclassified Bacteria | Unclassified Bacteria | Unclassified Bacteria | Unclassified Bacteria | 9.311 |
|  | Beech | Oxic | Proteobacteria | Betaproteobacteria | Burkholderiales | Comamonadaceae | Unclassified Comamonadaceae | 8.660 |
|  | Beech | Anoxic | Proteobacteria | Betaproteobacteria | Burkholderiales | Comamonadaceae | Unclassified Comamonadaceae | 5.848 |
|  | Beech | Oxic | Proteobacteria | Alphaproteobacteria | Sphingomonadales | Erythrobacteraceae | Novosphingobium | 5.773 |
|  | Beech | Anoxic | Proteobacteria | Betaproteobacteria | Burkholderiales | Comamonadaceae | Aquabacterium | 5.707 |
|  | Beech | Oxic | Proteobacteria | Betaproteobacteria | Nitrosomonadales | Methylophilaceae | Methylophilus | 5.659 |
|  | Maple | Oxic | Bacteroidetes | Flavobacteriia | Flavobacteriales | Flavobacteriaceae | Flavobacterium | 18.650 |
|  | Maple | Anoxic | Bacteroidetes | Flavobacteriia | Flavobacteriales | Flavobacteriaceae | Flavobacterium | 14.390 |
|  | Maple | Anoxic | Unclassified Bacteria | Unclassified Bacteria | Unclassified Bacteria | Unclassified Bacteria | Unclassified Bacteria | 12.819 |
|  | Maple | Oxic | Unclassified Bacteria | Unclassified Bacteria | Unclassified Bacteria | Unclassified Bacteria | Unclassified Bacteria | 12.475 |
|  | Maple | Anoxic | Proteobacteria | Gammaproteobacteria | Pseudomonadales | Moraxellaceae | Acinetobacter | 6.760 |
|  | Maple | Anoxic | Proteobacteria | Betaproteobacteria | Burkholderiales | Comamonadaceae | Unclassified Comamonadaceae | 6.111 |
|  | Maple | Oxic | Proteobacteria | Betaproteobacteria | Burkholderiales | Comamonadaceae | Unclassified Comamonadaceae | 5.922 |
| Faecal_pellets | Alder | Anoxic | Proteobacteria | Gammaproteobacteria | Pseudomonadales | Moraxellaceae | Acinetobacter | 16.048 |
|  | Alder | Oxic | Proteobacteria | Gammaproteobacteria | Pseudomonadales | Moraxellaceae | Acinetobacter | 13.495 |
|  | Alder | Oxic | Proteobacteria | Gammaproteobacteria | Enterobacterales | Erwiniaceae | Pantoea | 12.704 |
|  | Alder | Oxic | Proteobacteria | Gammaproteobacteria | Enterobacterales | Enterobacteriaceae | Klebsiella | 9.286 |
|  | Alder | Anoxic | Proteobacteria | Betaproteobacteria | Burkholderiales | Comamonadaceae | Unclassified Comamonadaceae | 8.387 |
|  | Alder | Oxic | Proteobacteria | Betaproteobacteria | Burkholderiales | Comamonadaceae | Unclassified Comamonadaceae | 5.912 |
|  | Alder | Anoxic | Unclassified Bacteria | Unclassified Bacteria | Unclassified Bacteria | Unclassified Bacteria | Unclassified Bacteria | 5.394 |
|  | Alder | Anoxic | Firmicutes | Bacilli | Lactobacillales | Carnobacteriaceae | Carnobacterium | 5.302 |
|  | Beech | Anoxic | Proteobacteria | Gammaproteobacteria | Pseudomonadales | Moraxellaceae | Acinetobacter | 34.836 |
|  | Beech | Oxic | Proteobacteria | Gammaproteobacteria | Pseudomonadales | Moraxellaceae | Acinetobacter | 19.049 |
|  | Beech | Oxic | Firmicutes | Bacilli | Lactobacillales | Carnobacteriaceae | Carnobacterium | 12.678 |
|  | Beech | Anoxic | Unclassified Bacteria | Unclassified Bacteria | Unclassified Bacteria | Unclassified Bacteria | Unclassified Bacteria | 11.787 |
|  | Beech | Anoxic | Firmicutes | Bacilli | Lactobacillales | Carnobacteriaceae | Carnobacterium | 11.125 |
|  | Beech | Oxic | Unclassified Bacteria | Unclassified Bacteria | Unclassified Bacteria | Unclassified Bacteria | Unclassified Bacteria | 11.031 |
|  | Beech | Oxic | Proteobacteria | Alphaproteobacteria | Sphingomonadales | Sphingomonadaceae | Sphingobium | 6.529 |
|  | Beech | Oxic | Bacteroidetes | Flavobacteriia | Flavobacteriales | Flavobacteriaceae | Unclassified Flavobacteriaceae | 5.439 |
|  | Maple | Anoxic | Proteobacteria | Gammaproteobacteria | Pseudomonadales | Moraxellaceae | Acinetobacter | 25.815 |
|  | Maple | Oxic | Proteobacteria | Gammaproteobacteria | Pseudomonadales | Moraxellaceae | Acinetobacter | 18.772 |
|  | Maple | Oxic | Firmicutes | Bacilli | Lactobacillales | Carnobacteriaceae | Carnobacterium | 9.961 |
|  | Maple | Oxic | Unclassified Bacteria | Unclassified Bacteria | Unclassified Bacteria | Unclassified Bacteria | Unclassified Bacteria | 9.529 |
|  | Maple | Anoxic | Firmicutes | Bacilli | Lactobacillales | Carnobacteriaceae | Carnobacterium | 9.446 |
|  | Maple | Oxic | Proteobacteria | Gammaproteobacteria | Enterobacterales | Erwiniaceae | Pantoea | 8.211 |
|  | Maple | Anoxic | Proteobacteria | Betaproteobacteria | Burkholderiales | Comamonadaceae | Unclassified Comamonadaceae | 6.569 |
|  | Maple | Oxic | Proteobacteria | Gammaproteobacteria | Enterobacterales | Enterobacteriaceae | Klebsiella | 6.393 |
|  | Maple | Anoxic | Bacteroidetes | Flavobacteriia | Flavobacteriales | Flavobacteriaceae | Unclassified Flavobacteriaceae | 5.655 |
|  | Maple | Anoxic | Unclassified Bacteria | Unclassified Bacteria | Unclassified Bacteria | Unclassified Bacteria | Unclassified Bacteria | 5.162 |

**Table. S8.** Alpha diversity indices are based on rarefaction method for FPOM types collected during feeding experiment. Mean and standard deviation for species richness (Observed), Chao1 and Shannon diversity are shown.

| **FPOM_type** | **Leaf species** | **Conditioning** | **Observed** | **Chao1** | **Shannon** |
| --- | --- | --- | --- | --- | --- |
| Initial_leaves | Alder | Anoxic | 86±9 | 123±30 | 3.4±0.2 |
|  |  | Oxic | 180±11 | 277±27 | 4.4±0.1 |
|  | Beech | Anoxic | 117±8 | 148±17 | 3.5±0.1 |
|  |  | Oxic | 166±4 | 276±26 | 4.0±0.2 |
|  | Maple | Anoxic | 96±6 | 130±28 | 3.4±0.1 |
|  |  | Oxic | 169±13 | 223±25 | 4.3±0.1 |
| Final_leaves | Alder | Anoxic | 199±36 | 328±88 | 4.2±0.3 |
|  |  | Oxic | 236±33 | 380±56 | 4.6±0.3 |
|  | Beech | Anoxic | 238±8 | 379±27 | 4.6±0.1 |
|  |  | Oxic | 240±6 | 392±70 | 4.8±0.1 |
|  | Maple | Anoxic | 205±7 | 335±51 | 4.4±0.1 |
|  |  | Oxic | 258±7 | 417±23 | 4.9±0.1 |
| Faecal_pellets | Alder | Anoxic | 146±11 | 256±37 | 3.9±0.2 |
|  |  | Oxic | 153±11 | 235±19 | 3.8±0.1 |
|  | Beech | Anoxic | 97±7 | 155±34 | 3.1±0.2 |
|  |  | Oxic | 116±4 | 240±49 | 3.3±0.3 |
|  | Maple | Anoxic | 134±7 | 250±13 | 3.6±0.2 |
|  |  | Oxic | 124±42 | 206±62 | 3.5±0.5 |

**Table S9.** Results of the linear model to observe the effects of leaf species, conditioning, FPOM type and their interactions on bacterial diversity associated to shredder-produced FPOM. The model structure is H` ~ leaf species*conditioning*FPOM type where alder treatment and faecal pellet for FPOM type was used as reference. Abbreviations; SE: Standard error, H`: Shannon-Wiener Index.

| **Factors** | ***Estimate*** | ***SE*** | ***t-value*** | ***p-values*** |
| --- | --- | --- | --- | --- |
| FPOM typeFinal_leaves | 0.36 | 0.17 | 2.06 | **<0.05** |
| FPOM typeInitial_leaves | -0.45 | 0.17 | -2.60 | **<0.05** |
| ConditioningOxic | -0.03 | 0.17 | -0.18 | 0.860 |
| TreatmentBeech | -0.79 | 0.17 | -4.51 | **<0.05** |
| TreatmentMaple | -0.26 | 0.17 | -1.47 | 0.151 |
| FPOM typeFinal_leaves:ConditioningOxic | 0.44 | 0.25 | 1.78 | 0.084 |
| FPOM typeInitial_leaves:ConditioningOxic | 1.00 | 0.25 | 4.03 | **<0.05** |
| FPOM typeFinal_leaves:TreatmentBeech | 1.17 | 0.25 | 4.73 | **<0.05** |
| FPOM typeInitial_leaves:TreatmentBeech | 0.83 | 0.25 | 3.36 | **<0.05** |
| FPOM typeFinal_leaves:TreatmentMaple | 0.40 | 0.25 | 1.63 | 0.112 |
| FPOM typeInitial_leaves:TreatmentMaple | 0.28 | 0.25 | 1.12 | 0.270 |
| ConditioningOxic:TreatmentBeech | 0.21 | 0.26 | 0.81 | 0.423 |
| ConditioningOxic:TreatmentMaple | -0.14 | 0.25 | -0.55 | 0.586 |
| FPOM typeFinal_leaves:ConditioningOxic:TreatmentBeech | -0.47 | 0.36 | -1.31 | 0.197 |
| FPOM typeInitial_leaves:ConditioningOxic:TreatmentBeech | -0.63 | 0.36 | -1.75 | 0.089 |
| FPOM typeFinal_leaves:ConditioningOxic:TreatmentMaple | 0.23 | 0.35 | 0.66 | 0.511 |
| FPOM typeInitial_leaves:ConditioningOxic:TreatmentMaple | 0.02 | 0.35 | 0.06 | 0.950 |

***Statistically significant p-values (< 0.05) values are shown in bold***

**Table S10.** Taxonomic classification of bacterial communities present in all sample types characterized by Indicator Species Analysis. Taxa associated to different groups with stat value above 0.90 are shown here. All stat values shown here are statistically significant (p < 0.05).

| **Sample_type** | **Phylum** | **Class** | **Order** | **Family** | **Genus** | **stat** |
| --- | --- | --- | --- | --- | --- | --- |
| Initial anoxic  leaf | Bacteroidetes | Bacteroidia | Bacteroidales | Paludibacteraceae | Paludibacter | 1.000 |
|  | Proteobacteria | Betaproteobacteria | Rhodocyclales | Rhodocyclaceae | Propionivibrio | 0.995 |
|  | Firmicutes | Clostridia | Clostridiales | Eubacteriaceae | Alkalibacter | 0.994 |
|  | Firmicutes | Clostridia | Clostridiales | Lachnospiraceae | Unclassified Lachnospiraceae | 0.993 |
|  | Firmicutes | Clostridia | Clostridiales | Natranaerovirga | Unclassified Natranaerovirga | 0.993 |
|  | Firmicutes | Clostridia | Clostridiales | Lachnospiraceae | Anaerotaenia | 0.992 |
|  | Proteobacteria | Gammaproteobacteria | Aeromonadales | Aeromonadaceae | Tolumonas | 0.992 |
|  | Proteobacteria | Gammaproteobacteria | Aeromonadales | Aeromonadaceae | Pseudaeromonas | 0.992 |
|  | Campilobacterota | Campylobacteria | Campylobacterales | Sulfurospirillaceae | Sulfurospirillum | 0.988 |
|  | Firmicutes | Negativicutes | Selenomonadales | Sporomusaceae | Sporomusa | 0.988 |
|  | Proteobacteria | Betaproteobacteria | Neisseriales | Chromobacteriaceae | Formivibrio | 0.987 |
|  | Proteobacteria | Gammaproteobacteria | Alteromonadales | Shewanellaceae | Shewanella | 0.980 |
|  | Proteobacteria | Gammaproteobacteria | Enterobacterales | Erwiniaceae | Unclassified Erwiniaceae | 0.978 |
|  | Firmicutes | Bacilli | Lactobacillales | Carnobacteriaceae | Trichococcus | 0.977 |
|  | Proteobacteria | Gammaproteobacteria | Enterobacterales | Pectobacteriaceae | Pectobacterium | 0.972 |
|  | Bacteroidetes | Bacteroidia | Bacteroidales | Prolixibacteraceae | Unclassified Prolixibacteraceae | 0.959 |
|  | Proteobacteria | Gammaproteobacteria | Aeromonadales | Aeromonadaceae | Aeromonas | 0.958 |
|  | Firmicutes | Clostridia | Clostridiales | Clostridiaceae_1 | Clostridium_sensu_stricto | 0.955 |
|  | Firmicutes | Clostridia | Clostridiales | Eubacteriaceae | Acetobacterium | 0.952 |
|  | Proteobacteria | Alphaproteobacteria | Rhizobiales | Beijerinckiaceae | Unclassified Beijerinckiaceae | 0.945 |
|  | Proteobacteria | Gammaproteobacteria | Pseudomonadales | Pseudomonadaceae | Pseudomonas | 0.942 |
|  | Bacteroidetes | Bacteroidia | Bacteroidales | Marinilabiliaceae | Unclassified Marinilabiliaceae | 0.942 |
|  | Proteobacteria | Betaproteobacteria | Neisseriales | Chromobacteriaceae | Iodobacter | 0.935 |
|  | Firmicutes | Clostridia | Clostridiales | Unclassified Clostridiales | Unclassified Clostridiales | 0.929 |
|  | Proteobacteria | Betaproteobacteria | Rhodocyclales | Zoogloeaceae | Uliginosibacterium | 0.926 |
|  | Proteobacteria | Alphaproteobacteria | Rhizobiales | Unclassified Rhizobiales | Unclassified Rhizobiales | 0.924 |
|  | Firmicutes | Negativicutes | Selenomonadales | Sporomusaceae | Pelosinus | 0.917 |
|  | Proteobacteria | Gammaproteobacteria | Enterobacterales | Pectobacteriaceae | Brenneria | 0.917 |
|  | Proteobacteria | Gammaproteobacteria | Enterobacterales | Enterobacteriaceae | Buttiauxella | 0.915 |
|  | Proteobacteria | Alphaproteobacteria | Rhodospirillales | Acetobacteraceae | Unclassified Acetobacteraceae | 0.914 |
|  | Proteobacteria | Alphaproteobacteria | Rhizobiales | Methylobacteriaceae | Methylobacterium | 0.908 |
| All other leaves | Bacteroidetes | Flavobacteriia | Flavobacteriales | Flavobacteriaceae | Flavobacterium | 1.00 |
|  | Unclassified Bacteria | Unclassified Bacteria | Unclassified Bacteria | Unclassified Bacteria | Unclassified Bacteria | 0.981 |
|  | Firmicutes | Clostridia | Clostridiales | Unclassified Clostridiales | Unclassified Clostridiales | 0.975 |
|  | Proteobacteria | Gammaproteobacteria | Pseudomonadales | Pseudomonadaceae | Pseudomonas | 0.974 |
|  | Proteobacteria | Alphaproteobacteria | Sphingomonadales | Erythrobacteraceae | Novosphingobium | 0.963 |
|  | Proteobacteria | Gammaproteobacteria | Cellvibrionales | Cellvibrionaceae | Cellvibrio | 0.958 |
|  | Proteobacteria | Betaproteobacteria | Burkholderiales | Oxalobacteraceae | Massilia | 0.955 |
|  | Proteobacteria | Alphaproteobacteria | Caulobacterales | Caulobacteraceae | Asticcacaulis | 0.944 |
|  | Bacteroidetes | Sphingobacteriia | Sphingobacteriales | Sphingobacteriaceae | Pedobacter | 0.935 |
|  | Proteobacteria | Alphaproteobacteria | Rhizobiales | Rhizobiaceae | Neorhizobium | 0.918 |
|  | Proteobacteria | Betaproteobacteria | Burkholderiales | Oxalobacteraceae | Duganella | 0.910 |
|  | Bacteroidetes | Sphingobacteriia | Sphingobacteriales | Sphingobacteriaceae | Pedobacter | 0.909 |
|  | Proteobacteria | Betaproteobacteria | Burkholderiales | Comamonadaceae | Rhizobacter | 0.907 |
|  | Proteobacteria | Alphaproteobacteria | Caulobacterales | Caulobacteraceae | Caulobacter | 0.902 |
| Faecal pellets | Actinobacteria | Actinobacteria | Unclassified Actinobacteria | Unclassified Actinobacteria | Unclassified Actinobacteria | 1.000 |
|  | Proteobacteria | Gammaproteobacteria | Pseudomonadales | Moraxellaceae | Acinetobacter | 0.999 |
|  | Firmicutes | Unclassified Firmicutes | Unclassified Firmicutes | Unclassified Firmicutes | Unclassified Firmicutes | 0.997 |
|  | Proteobacteria | Gammaproteobacteria | Gammaproteobacteria_incertae_sedis | Unclassified Gammaproteobacteria_incertae_sedis | Unclassified Gammaproteobacteria_incertae_sedis | 0.996 |
|  | Firmicutes | Bacilli | Lactobacillales | Carnobacteriaceae | Carnobacterium | 0.995 |
|  | Bacteroidetes | Flavobacteriia | Flavobacteriales | Flavobacteriaceae | Unclassified Flavobacteriaceae | 0.992 |
|  | Actinobacteria | Actinobacteria | Micrococcales | Unclassified Micrococcales | Unclassified Micrococcales | 0.992 |
|  | Actinobacteria | Actinobacteria | Micrococcales | Microbacteriaceae | Leucobacter | 0.992 |
|  | Firmicutes | Bacilli | Lactobacillales | Enterococcaceae | Vagococcus | 0.990 |
|  | Actinobacteria | Actinobacteria | Micrococcales | Microbacteriaceae | Subtercola | 0.987 |
|  | Proteobacteria | Alphaproteobacteria | SAR11 | Candidatus_Pelagibacter | Unclassified Candidatus_Pelagibacter | 0.987 |
|  | Deinococcus-Thermus | Deinococci | Deinococcales | Deinococcaceae | Deinococcus | 0.974 |
|  | Actinobacteria | Actinobacteria | Mycobacteriales | Nocardiaceae | Rhodococcus | 0.973 |
|  | Proteobacteria | Betaproteobacteria | Burkholderiales | Burkholderiaceae | Formosimonas | 0.968 |
|  | Proteobacteria | Betaproteobacteria | Neisseriales | Neisseriaceae | Unclassified Neisseriaceae | 0.968 |
|  | Proteobacteria | Gammaproteobacteria | Pseudomonadales | Moraxellaceae | Enhydrobacter | 0.967 |
|  | Bacteroidetes | Bacteroidia | Bacteroidales | Unclassified Bacteroidales | Unclassified Bacteroidales | 0.966 |
|  | Actinobacteria | Actinobacteria | Micrococcales | Microbacteriaceae | Cryobacterium | 0.965 |
|  | Proteobacteria | Alphaproteobacteria | Caulobacterales | Caulobacteraceae | Brevundimonas | 0.964 |
|  | Proteobacteria | Gammaproteobacteria | Gammaproteobacteria_incertae_sedis | Ignatzschineria | Unclassified Ignatzschineria | 0.957 |
|  | Proteobacteria | Alphaproteobacteria | Sphingomonadales | Sphingomonadaceae | Sphingomonas | 0.956 |
|  | Proteobacteria | Betaproteobacteria | Unclassified Betaproteobacteria | Unclassified Betaproteobacteria | Unclassified Betaproteobacteria | 0.940 |
|  | Proteobacteria | Gammaproteobacteria | Pseudomonadales | Moraxellaceae | Alkanindiges | 0.934 |
|  | Proteobacteria | Betaproteobacteria | Burkholderiales | Comamonadaceae | Unclassified Comamonadaceae | 0.934 |
|  | Acidobacteria | Blastocatellia | Blastocatellales | Blastocatellaceae | Stenotrophobacter | 0.925 |
|  | Unclassified Bacteria | Unclassified Bacteria | Unclassified Bacteria | Unclassified Bacteria | Unclassified Bacteria | 0.920 |
|  | Actinobacteria | Actinobacteria | Micrococcales | Microbacteriaceae | Unclassified Microbacteriaceae | 0.907 |
